# Muscle, season, sex, and carcass weight affected pork texture, collagen characteristics, and intramuscular fat content

**DOI:** 10.1093/jas/skae231

**Published:** 2024-08-23

**Authors:** Xiying Li, Minh Ha, Robyn D Warner, Amy Lealiifano, Robert J E Hewitt, Darryl N D’Souza, Megan Trezona, Frank R Dunshea

**Affiliations:** School of Agriculture, Food and Ecosystem, Faculty of Science, The University of Melbourne, Parkville VIC 3010, Australia; School of Agriculture, Food and Ecosystem, Faculty of Science, The University of Melbourne, Parkville VIC 3010, Australia; School of Agriculture, Food and Ecosystem, Faculty of Science, The University of Melbourne, Parkville VIC 3010, Australia; Rivalea (Australia) Pty Ltd, JBS Australia Pork Division, Corowa, NSW 2646, Australia; SunPork Group, Eagle Farm, QLD 4009, Australia; SunPork Group, Eagle Farm, QLD 4009, Australia; Linley Valley Pork, Wundowie, WA 6560, Australia; School of Agriculture, Food and Ecosystem, Faculty of Science, The University of Melbourne, Parkville VIC 3010, Australia; Faculty of Biological Sciences, University of Leeds, Leeds LS2 9JT, UK

**Keywords:** pork quality, Warner–Bratzler shear force, texture profile analysis, pH, correlation

## Abstract

In this study, pigs from 3 supply chains were slaughtered in an Australian summer and winter (*n* = 20 for each supply chain). The pigs were from 2 sexes (female and castrated male) and 2 carcass weight groups (high: 95.0 to 100.0 kg and low: 75.0 to 80.0 kg). From each carcass, the *Biceps femoris* (BF), *Longissimus thoracis et lumborum* (LTL), and *Triceps brachii* (TB) were excised at 24 h postmortem, vacuum packed, frozen at 24-48 h and transported to the lab. Cooking loss, Warner–Bratzler shear force (WBSF) and texture profile analysis (adhesiveness, chewiness, cohesiveness, hardness, resilience, and springiness) were measured in LTL and BF. pH, collagen content, and solubility and intramuscular fat (IMF) content were determined for all muscles. Results showed that BF was tougher than LTL, and winter samples were tougher than summer ones (*P* < 0.05). The TB had higher pH, collagen, and IMF content than BF and LTL (*P* < 0.05). Collagen solubility was higher in castrated male and winter samples. pH, collagen solubility, and IMF content were significantly (*P* < 0.05) related to chewiness and hardness in pork BF and LTL. pH and IMF were also related to cooking loss, while collagen solubility and IMF were related to WBSF (*P* < 0.05). The relationships of pH and IMF with pork texture were predominantly driven by the LTL, while the relationships between collagen solubility and texture were predominantly driven by the BF. Collagen solubility and IMF of pork BF and TB were related to those of LTL, but the correlations were not strong enough for prediction. Pork texture and chemical components were affected by muscle, seasons, sex and carcass weight. pH, collagen solubility, and IMF-affected pork texture.

## Teaser

Pork texture and chemical components were affected by muscle, season, sex and carcass weights. pH, collagen solubility, and intramuscular fat content were positively related to pork instrumental tenderness.

## Introduction

Meat quality is affected by various production factors and muscle types. Jeleníková et al. (2008) reported that pork from female pigs have higher intramuscular fat (IMF) content and receive higher tenderness scores in sensory evaluation than castrated male, while Gispert et al. (2010) reported that IMF of *Semimembranosus* (SM) from immunocastrated male pigs was higher than that of female pigs. Čandek-Potokar et al. (1998) found that heavier pigs produced meat with higher IMF content. Judge et al. (1959) reported that pigs raised and slaughtered in cooler seasons produced more highly marbled and darker loin muscles than those raised in warmer seasons. However, the effects of season on the texture and chemical properties of pork were less studied, and the control of climate within the sheds was improved since 1959. In addition, a number of studies on the effects of sex, season and carcass weight on pork quality only focussed on the loin muscle (Judge et al., 1959; Čandek-Potokar et al., 1998; Bañón et al., 2004), but this is only one muscle in the carcass and only a few studies investigated the relationship between meat quality of the loin and other muscles. The *Triceps brachii* (TB) has a higher collagen content than *Longissimus thoracis et lumborum* (LTL), but it was preferred by consumers for several sensory attributes compared to the LTL (Wheeler et al., 2000; Channon et al., 2016). Previously, Li et al. (2022a) found that *Biceps femoris* (BF) was tougher and had higher collagen content than LTL. Nevertheless, few studies have been conducted on the chemical properties of pork TB and its comparison with LTL and BF. Kim et al. (2008) found that BF had similar IMF content compared to TB, and was higher than that of LTL, but they did not measure collagen characteristics.

The effects of pH, collagen, and IMF on meat texture have been extensively studied. pH affected meat toughness. The curve of Warner–Bratzler shear force (WBSF) vs ultimate pH has been plotted in several studies (Purchas, 1990; Devine et al., 1993; Watanabe et al., 1996), indicating that there is a curvilinear relationship with WBSF decreasing as pH increases up to pH 6.2. For collagen content and solubility, contradictory findings were reported for the effects of collagen on meat texture (Wheeler et al., 2002; Jeremiah et al., 2003; Christensen et al., 2011; Li et al., 2022b). A previous meta-analysis showed that collagen content and solubility were correlated positively and negatively, respectively, with WBSF in beef, especially in muscles other than loin. However, no significant relationship was found in pork possibly because of the limited number of studies (Li et al., 2022b). Similar to collagen, the effect of IMF on meat texture is also debatable, especially at the low IMF observed in improved genotypes. Significant relationships were found between IMF content and meat texture in some studies, but they varied among muscles and genetics (Laack et al., 2001; Rincker et al., 2008; Starkey et al., 2016). More studies are needed to understand the effects of pH, collagen and IMF on pork texture and to conduct a meta-analysis on pork to find out general relationship between chemical properties and pork texture.

The meat industry has been interested in predicting the quality of different pork muscles from parameters measured in the LTL. Font-i-Furnols et al. (2019) found that the IMF content of pork BF, SM, and *Gluteus medius* was correlated with the IMF of the LTL. The authors explained that when IMF increased, it increased for all muscles (Font-i-Furnols et al., 2019). However, Arkfeld et al. (2016) and Huff-Lonergan et al. (2002) reported that pH and colour of SM were weakly correlated with those of LTL. More studies are required to investigate whether the LTL can be used as a reference muscle to predict the quality of other muscles.

Therefore, this study aims to 1) investigate the effects of muscle, season, sex and carcass weight on pork texture, collagen characteristics, and IMF content; 2) study the influence of pH, collagen and IMF on pork texture; 3) investigate whether the quality of BF and TB can be predicted by that of LTL. We hypothesized that 1) IMF of BF and TB was higher than that of LTL and BF was tougher than LTL; 2) pork with higher hot carcass weight were more tender and had higher IMF content; 3) castrated male had higher IMF content than female pigs, while pigs raised in winter had higher IMF content; 4) pH, collagen, and IMF affected pork texture, especially in the BF; 5) the texture and chemical properties of BF and TB were correlated with those of LTL.

## Materials and Methods

### Sample preparation

Pigs from 3 supply chains were slaughtered (A, B, and C) in an Australian summer (February) and winter (August, *n* = 10 per supply chain in one season). All slaughterhouses followed the Australian Standard AS 4696:2007 Hygienic Production and Transportation of Meat and Meat Products for Human Consumption, Industry Animal Welfare Standards for Livestock Processing Establishments Preparing Meat for Human Consumption—Third Edition and Model Code of Practice for the Welfare of Animals: Livestock at Slaughtering Establishments. Within each supply chain in one season, pigs were from 2 hot carcass weight groups: high (95.0 to 100.0 kg) and low (75.0 to 80.0 kg), and 2 sex groups (castrated male and female), giving four combinations: castrated male in low weight group (*n* = 2), castrated male in high weight group (*n* = 3), female in low weight group (*n* = 3) and female in high weight group (*n* = 2). Supply chain A used physical castration at 4 d of age, while supply chains B and C used Improvac castration (Dunshea et al., 2001). All pigs sourced for this study were commercial slaughter pigs from crossbred lines. Pigs from supply chain A (composite line, Large White × Landrace × Duroc) were raised on a large commercial indoor farm, farrow to finish site. Sheds were environmentally controlled, tunnel ventilated, and flooring is concrete slats. Pigs were fed ad libitum with commercial, pelleted, cereal-based diets on a phase feeding program (5 diets wean-finisher). Pigs from supply chain B (Large White × Landrace) were raised on a large commercial piggery. All pigs were housed indoors with slatted floors. They were fed ad libitum with commercial pelleted diets. Pigs from supply chain C (Large White × Landrace × Duroc) were raised on a large commercial piggery. All pigs were housed indoors with slatted floors. They were fed ad libitum with commercial pelleted diets.

From each pig, the *Biceps femoris* (BF), *Longissimus thoracis et lumborum* (LTL), and *Triceps brachii* (TB) were excised at 24 h post-mortem, vacuum packed, frozen at 24-48 h post-mortem (supply chain A froze at 24 h, supply chain B froze at 28 h and supply chain C froze at 48 h) and transported to The University of Melbourne. One TB from winter was missing, resulting in a total number of 179 muscles.

Samples were kept frozen at −20 °C until analysis. Samples were cut frozen using a meat and bone bench band saw (CARNIVORE equipment, Melbourne, Australia). Samples of 75 g were stored in sample jars and kept frozen before being freeze-dried for 72 h. From BF and LTL, a cube of around 5.0 × 5.0 × 5.0 cm^3^ weighing 110.0 ± 10.0 g was cut, vacuum packed and kept frozen for cooking loss and texture analysis. No texture analysis was performed for TB due to sample size constraints since these muscles were also being used for sensory analysis (to be reported separately). A small piece from each muscle was removed, thawed, and used for pH measurement using a portable pH/temperature metre (TPS WP-80M, Brendale, QLD, Australia) equipped with an electrode (TPS-ionode, Niddrie, VIC, Australia).

### Cooking loss, Warner–Bratzler shear force and texture profile analysis

Pork BF and LTL were cooked from frozen in vacuum-packed pouches in a circulating water bath (JULABO GmbH, Seelbach, Germany) at 75°C. Internal temperature was monitored by a temperature probe (JULABO GmbH, Seelbach, Germany) inserted into a control sample with the same treatment. Samples were removed when the internal temperature reached 70 °C and the samples were placed in iced water for 30 min. Seventy degreeCelcius was chosen because of the cooking instruction recommended for pork sensory evaluation (Channon et al., 2016). The samples were then stored in a cold room at 2 °C overnight. Samples were allocated to cooking batches based on supply chain and muscles with 10 samples per batch. On the following day, muscles were taken out from the package, dried with paper towel, and weighed. Cooking loss was calculated based on the weight difference before and after cooking.

Warner–Bratzler shear force (WBSF) and texture profile analysis (TPA) were measured as described by Li et al. (2022b). Briefly, six cuboids of 1.0 × 1.0 × 2.5 cm^3^ were cut from each muscle for WBSF measurement with fibres parallel to the long axis. The measurement was performed with a V-shape blade using a Lloyd Texture Analyser TA2 (AMETEK, Berwyn, Pennsylvania, USA). For TPA, a steak of 1.0-cm thick was cut and a cylindrical probe with a diameter of 6 mm was used to compress the sample to 80% of its height. Texture profile was obtained in a double bite process. Adhesiveness (Nmm), cohesiveness, chewiness (N), hardness (N), resilience, and springiness were obtained using the NEXYGENPlus program (AMETEK, Berwyn, Pennsylvania, USA).

### Collagen content and solubility

Collagen content and solubility measurements followed the AOAC method 990.26 (Kolar, 1990) for hydroxyproline with some modifications (Li et al., 2022a). Briefly, freeze-dried meat was powdered with a knife and a chopping board by chopping the freeze-dried meat into small particles. For total collagen content, triplicates of 0.2 g freeze-dried powder were hydrolysed in 3.5 M H_2_SO_4_ for 16 h at 105 °C. For soluble collagen, 1.5 g of freeze-dried powder was added to 10 ml of water and heated at 80 °C for 2 h with vortexing every 30 min. After being centrifuged at 4,750 × *g* for 30 min, duplicates of 0.5 ml supernatant were used for acid hydrolysis. After dilution, pH adjustment, oxidizing and colour reaction, hydroxyproline content was determined by measuring absorbance at 558 nm. The standard curve was prepared with hydroxyproline at 0, 1.2, 2.4, 3.6, 4.8, and 6.0 µg/ml. A conversion factor of 7.25 was applied to calculate collagen content from hydroxyproline content. Collagen content was expressed as mg collagen per g of fresh meat, while collagen solubility was the percentage of soluble collagen over total collagen content.

### Intramuscular fat content

Intramuscular fat (IMF) content was determined by AOAC method 991.36 (AOAC, 1995) using Soxhlet extraction with some modifications. Triplicates of 3.5 g powdered freeze-dried samples were wrapped in Whatman no.1 filter paper before being put in the Soxhlet apparatus. The extraction lasted for 2 h with diethyl ether as solvent. The solvent was evaporated with a rotary evaporator followed by oven drying. The IMF content was expressed as the percentage of IMF in fresh meat.

### Statistical analysis

The effects of muscle, sex, carcass weight, and season on pork texture and chemical components were analysed in GenStat (16th Edition, VSN International, UK) using restricted maximum likelihood (REML) linear mixed models. The fixed terms were muscle + season + sex + weight group + Muscle × Season + Muscle × Sex + Muscle × Weight_group + Season × Weight_group + Season × Sex + Sex × Weight_group and the random term was supply chain. Surgical castrated and immunocastrated male pigs were grouped as castrated male. Surgically castration effect was mixed with farm effect, so surgical castration effect was not considered. The effects of chemical measurements on the texture and cooking loss of pork were analysed using a linear model and the model was *y* = Constant + Collagen content + Collagen solubility + IMF + pH + Muscle + Season + Sex + Weight group. Supply chain was the random term. This was performed on BF and LTL together as well as separately on BF or LTL. The slope and *P* value were recorded. Principal component analysis (PCA) was performed on combined LTL and BF as well as the muscle separately in RStudio (Posit, PBC, Boston, USA) using correlation matrix. Pearson’s correlation matrices between texture and chemical measurements as well as between BF and LTL were also performed.

## Results

Cooking loss and texture differed between muscles and seasons (Table 1). The BF showed higher cooking loss (*P* < 0.001), chewiness (*P* < 0.001), cohesiveness (*P* < 0.001), hardness (*P* < 0.001), resilience (*P* < 0.001) and springiness (*P* < 0.001) compared to the LTL. However, they did not differ in adhesiveness or WBSF. As for the seasonal difference, samples from winter had higher cooking loss (*P* < 0.001), chewiness (*P *= 0.009), hardness (*P *= 0.011) and springiness (*P *= 0.021) compared to those from summer. There was no difference between sex or weight groups (Table 1). There was a significant muscle × sex interaction for cohesiveness (*P* = 0.034) with the BF from castrated male had the highest cohesiveness. Within LTL, springiness of high weight group was higher than that of low weight group (0.798 vs 0.770, *P* = 0.038), but there was no difference in BF.

**Table 1.** Effects of muscle, season, sex and weight group on cooking loss and texture of pork

	Muscle (M)^1^			Season (S)			Weight group (W)			Sex			*P*-value^4^			
	BF	LTL	sed^2^	Winter	Summer	sed	High	Low	sed	Castrate	Female	sed	M	S	W	Sex
*n*	60	60		60	60		60	60		58	62					
Cooking loss (%)	23.0	19.6	0.468	22.1	20.5	0.468	21.0	21.6	0.47	21.4	21.2	0.47	**<0.001**	**<0.001**	0.17	0.88
Adhesiveness (Nmm)	11.6	10.8	0.56	11.3	11.1	0.56	11.6	10.7	0.56	11.0	11.3	0.56	0.15	0.76	0.097	0.78
Chewiness (N)	15.7	12.5	0.45	14.7	13.6	0.45	14.4	13.9	0.45	14.0	14.3	0.45	**<0.001**	**0.009**	0.32	0.53
Cohesiveness	0.456	0.431	0.0047	0.443	0.445	0.0047	0.445	0.443	0.0048	0.448	0.440	0.0048	**<0.001**	0.67	0.58	0.063
Hardness (N)	41.6	36.7	1.05	40.5	37.8	1.05	39.5	38.9	1.05	38.6	39.7	1.05	**<0.001**	**0.011**	0.60	0.30
Resilience	0.465	0.432	0.0054	0.451	0.446	0.0054	0.449	0.447	0.0054	0.450	0.446	0.0054	**<0.001**	0.37	0.77	0.48
Springiness	0.830	0.785	0.0092	0.818	0.797	0.0092	0.812	0.804	0.0092	0.801	0.815	0.0092	**<0.001**	**0.021**	0.36	0.15
WBSF^3^ (N)	35.6	33.4	1.32	34.6	34.4	1.32	33.7	35.3	1.33	34.1	34.4	1.32	0.11	0.87	0.21	0.50

^1^BF, *Biceps femoris*; LTL, *Longissimus thoracis et lumborum*.

^2^sed = standard error of difference.

^3^WBSF, Warner–Bratzler shear force.

^4^Fixed term = muscle + season + sex + weight group. Random term = supplier. Bold values were significant (*P* < 0.05).

Table 2 showed the chemical properties of the pork samples. pH of TB was the highest, followed by BF, and LTL was the lowest (*P* < 0.001). The same trend was observed for collagen content (TB > BF > LTL, *P* < 0.001). The TB had higher IMF content than BF and LTL (*P* < 0.001). Pork obtained during summer tended to have higher collagen content than those from winter (*P *= 0.056), while pork from winter had higher collagen solubility than summer samples (*P* < 0.001). Collagen solubility was higher in castrated male than female pork (*P* = 0.006). Also, IMF content of pork from the high-weight group tended to be higher than those from low-weight group and the difference was close to significant (*P *= 0.054).

**Table 2.** Effects of muscle, season and weight group on pH, collagen characteristics, and intramuscular fat content of pork

	Muscle (M)^1^				Season (S)			Weight group (W)^2^			Sex			*P*-value^4^			
	BF	LTL	TB	sed^3^	Winter	Summer	sed	High	Low	sed	Castrate	Female	sed	M	S	W	Sex
n	60	60	59		89	90		90	89		87	92					
pH	5.77^b^	5.67^c^	5.92^a^	0.023	5.77	5.80	0.019	5.80	5.77	0.019	5.78	5.80	0.019	**<0.001**	0.12	0.089	0.31
Collagen content (mg/g)	5.88^b^	4.07^c^	7.31^a^	0.201	5.60	5.91	0.164	5.64	5.87	0.165	5.86	5.65	0.165	**<0.001**	0.056	0.16	0.24
Collagen solubility (%)	5.77	6.44	6.05	0.283	7.00	5.18	0.231	6.16	6.01	0.232	6.40	5.77	0.232	0.058	**<0.001**	0.52	**0.006**
IMF^4^ (%)	1.18^b^	1.08^b^	2.43^a^	0.095	1.57	1.55	0.078	1.64	1.49	0.078	1.55	1.58	0.078	**<0.001**	0.72	0.054	0.83

^1^BF = *Biceps femoris*, LTL = *Longissimus thoracis et lumborum*, TB = *Triceps Brachii*.

^2^High hot carcass weight = 95.0 – 100.0 kg, low hot carcass weight = 75.0 – 80.0 kg.

^3^sed = standard error of difference.

^4^Fixed term = muscle + season + sex + weight group. Random term = supplier. Bold values were significant (*P* < 0.05).

^a, b, c^Data with different superscripts differ significantly between muscles.

**Figure 1.**
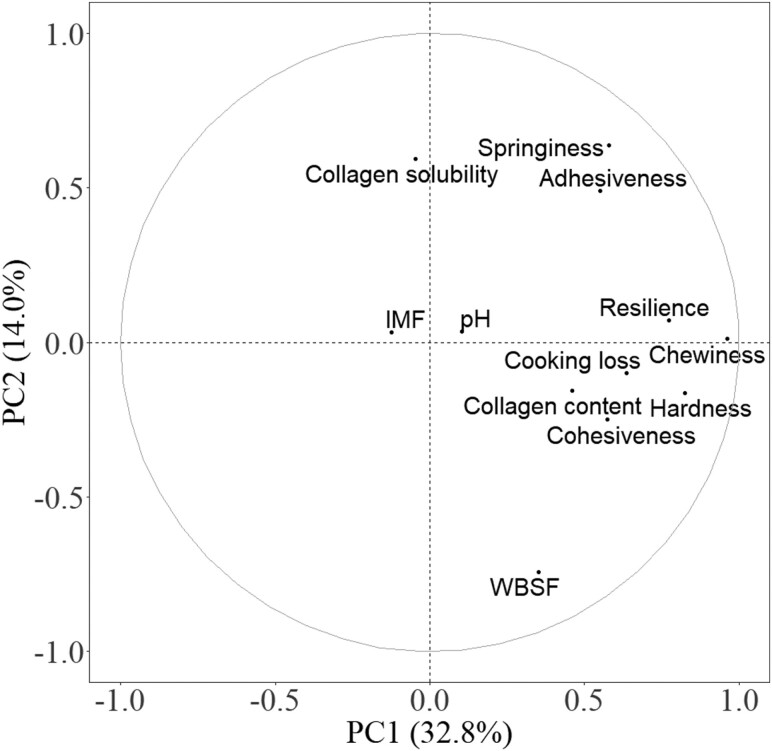
Principal component analysis (PCA) plot of texture parameters and chemical component of pork *Longissimus thoracis et lumborum* and *Biceps femoris*. IMF, intramuscular fat; WBSF, Warner–Bratzler shear force.

Figure 1 shows the PCA plot of texture and chemical measurements of both LTL and BF. PC1 explained 32.8% of the variation, while PC2 explained 14.0% of the variation. Collagen content was positively related to cooking loss, hardness, cohesiveness, and chewiness. Collagen solubility was negatively related to WBSF. Correlation matrices showed that IMF was negatively correlated (*P* < 0.05) with hardness, mainly in the LTL but not in the BF (Table 3).

**Table 3.** Correlation coefficients (Pearson’s *r*) between texture and muscle composition of both and individual muscle (BF, *Biceps femoris*; LTL, *Longissimus thoracis et lumborum*)

	Cooking loss	WBSF	Hardness	Cohesiveness	Adhesiveness	Chewiness	Resilience	Springiness
				Both				
Collagen content	0.34***	0.05	0.23^*^	0.31***	0.04	0.33***	0.32***	0.23^*^
Collagen solubility	0.04	−0.34***	−0.03	−0.08	0.05	−0.02	0.03	0.13
IMF	−0.08	−0.15	−0.21^*^	0.01	0.01	−0.12	−0.02	0.03
pH	−0.14	0.01	−0.02	0.04	0.07	0.07	0.05	−0.14
				BF				
Collagen content	−0.12	−0.17	−0.07	−0.01	−0.02	−0.06	0.03	0.02
Collagen solubility	0.22	−0.36**	−0.23	−0.12	−0.1	−0.22	−0.08	0.02
IMF	−0.05	−0.14	−0.1	0.07	0.12	−0.02	0.06	0.15
pH	−0.40**	−0.02	−0.01	−0.12	0.2	0.03	−0.13	0.16
				LTL				
Collagen content	0.01	0	−0.1	−0.07	−0.21	−0.17	−0.21	−0.25
Collagen solubility	0.13	−0.30^*^	0.27^*^	0.14	0.28^*^	0.36**	0.32^*^	0.43***
IMF	−0.26^*^	−0.2	−0.41**	−0.14	−0.15	−0.37**	−0.19	−0.16
pH	−0.38**	−0.07	−0.32^*^	−0.16	−0.19	−0.30^*^	−0.17	−0.06

^*^ *P* < 0.05, **P < 0.01, ***P < 0.001.

WBSF, Warner-Bratzler shear force; IMF, intramuscular fat.

**Figure 2.**
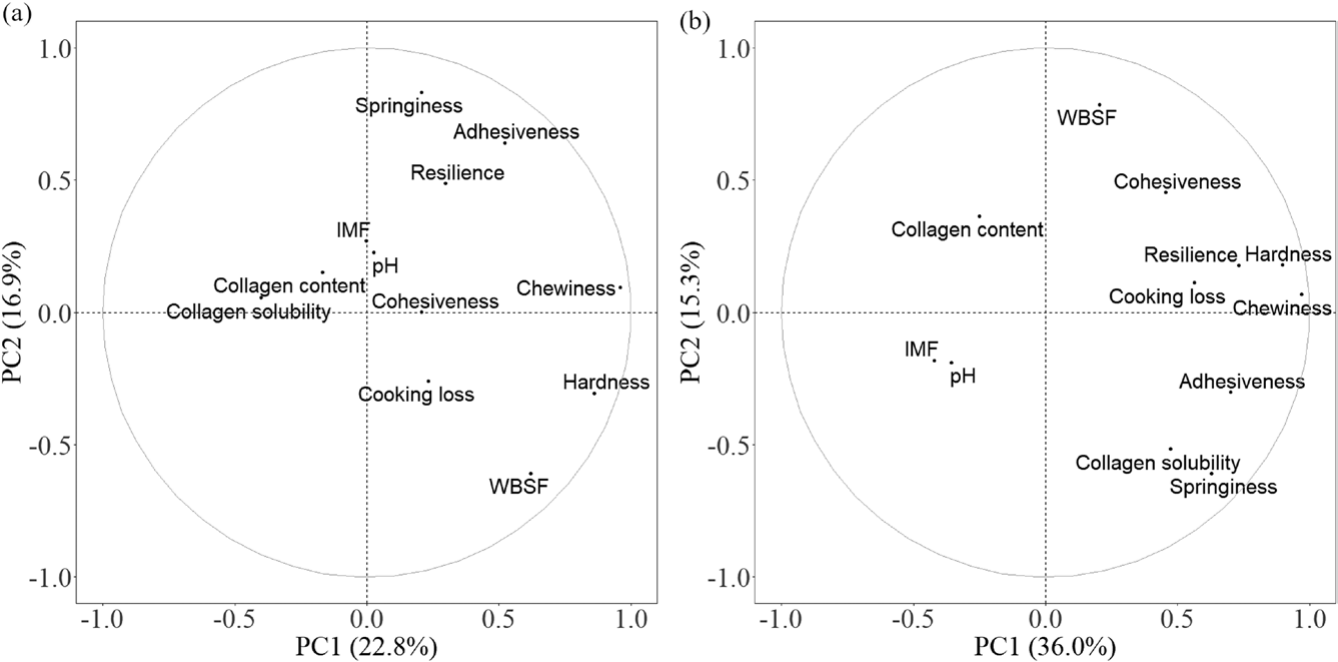
Principal component analysis (PCA) plot of texture parameters and chemical components of pork (a) *Biceps femoris* and (b) *Longissimus thoracis et lumborum*. IMF, intramuscular fat; WBSF, Warner–Bratzler shear force.

In the PCA plot of BF, PC1 explained 22.8% of the variation and PC2 explained 16.9% of the variation (Figure 2a). The contribution of chemical components in both PCs was low (contribution < 0.50). Collagen solubility was negatively correlated (*P* < 0.05) with WBSF and pH was negatively correlated with cooking loss (Table 3), but the PCA plot showed that pH had a small influence. In PCA plot of the LTL, PC1 and PC2 explained 36.0% and 15.3% of the variations, respectively (Figure 2b). Collagen solubility was positively related to springiness. IMF and pH were inversely related to cooking loss, hardness, chewiness and resilience.

When including other factors in the model, collagen content was not related to chewiness, hardness, WBSF, or cooking loss (Table 4). In both muscles, pH was negatively correlated with chewiness (*P* = 0.023), hardness (*P* = 0.006), and cooking loss (*P* < 0.001). Collagen solubility was negatively related to hardness (*P *= 0.042) and WBSF (*P* < 0.001), while IMF was negatively related to chewiness (*P *= 0.002), hardness (*P* < 0.001), WBSF (*P* < 0.001), and cooking loss (*P* = 0.009). In the BF, pH was related to cooking loss (*P* < 0.001). Collagen solubility was related to chewiness (*P* = 0.003), hardness (*P* < 0.001). WBSF (*P* < 0.001), and IMF was related to WBSF (*P* = 0.006). In the LTL, pH was related to chewiness (*P* = 0.009), hardness (*P* = 0.006), and cooking loss (*P* = 0.002). Collagen solubility was related to WBSF (*P* = 0.012), while IMF was related to chewiness (*P* = 0.005), hardness (*P* = 0.002), WBSF (*P* = 0.018), and cooking loss (*P* = 0.004).

**Table 4.** Effects of chemical measurements on texture and cooking loss of different pork muscles (BF, *Biceps femoris*; LTL, *Longissimus thoracis et lumborum*)

		Chewiness (N)		Hardness (N)		WBSF^1^ (N)		Cooking loss (%)	
		Slope^2^	*P*-value^3^	Slope	*P*-value	Slope	*P*-value	Slope	*P*-value
pH	Both	−4.00 ± 1.74	**0.023**	−11.1 ± 3.93	**0.006**	−5.75 ± 4.49	0.20	−8.99 ± 1.84	**<0.001**
	BF	−1.44 ± 2.41	0.55	−7.34 ± 5.59	0.195	−10.6 ± 6.27	0.096	−10.1 ± 2.86	**<0.001**
	LTL	−6.55 ± 2.41	**0.009**	−15.6 ± 5.46	**0.006**	−3.69 ± 6.33	0.56	−7.97 ± 2.50	**0.002**
Collagen content (mg/g)	Both	−0.240 ± 0.290	0.41	−0.440 ± 0.655	0.50	−1.27 ± 0.749	0.093	−0.185 ± 0.307	0.55
	BF	0.080 ± 0.325	0.81	0.240 ± 0.754	0.75	−0.422 ± 0.845	0.62	−0.271 ± 0.385	0.49
	LTL	−0.457 ± 0.548	0.41	−0.91 ± 1.24	0.46	−2.79 ± 1.43	0.057	−0.277 ± 0.566	0.63
Collagen solubility (%)	Both	−0.186 ± 0.179	0.30	−0.832 ± 0.404	**0.042**	−2.56 ± 0.462	**<0.001**	**−**0.103** **± 0.189	0.59
	BF	−0.730 ± 0.235	**0.003**	−2.02 ± 0.544	**<0.001**	−3.33 ± 0.610	**<0.001**	0.240 ± 0.278	0.39
	LTL	0.308 ± 0.275	0.27	0.292 ± 0.622	0.64	−1.89 ± 0.721	**0.012**	−0.560** **± 0.285	0.054
IMF^4^ (%)	Both	−1.66 ± 0.531	**0.002**	−4.91 ± 1.20	**<0.001**	−5.10 ± 1.37	**<0.001**	**−**1.50** **± 0.563	**0.009**
	BF	−0.714 ± 0.725	0.33	−3.27 ± 1.68	0.057	−5.38 ± 1.88	**0.006**	−0.972 ± 0.858	0.26
	LTL	−2.15 ± 0.742	**0.005**	−5.60 ± 1.68	**0.002**	−4.73 ± 1.94	**0.018**	−2.30 ± 0.767	**0.004**
R^2^ of the model	Both	32.6		23.1		22.4		41.8	
	BF	11.1		20.8		36.7		16.5	
	LTL	24.5		23.4		18.0		30.3	
RMSE^5^ of the model	Both	2.46		5.57		6.38		2.61	
	BF	2.24		5.18		5.81		2.65	
	LTL	2.46		5.56		6.44		2.54	

^1^Warner–Bratzler shear force.

^2^Slope was expressed as mean ± standard error.

^3^Model: *y* = Constant + Collagen content + Collagen solubility + IMF + pH + Muscle + Season + Sex + Weight group. Random factor = supplier. Bold values were significant (*P* < 0.05).

^4^intramuscular fat.

^5^root mean square error.

Cooking loss and hardness of BF were correlated (*P* < 0.05) with that of LTL (Table 5). pH and collagen content were only correlated (*P* < 0.05) between BF and LTL. Collagen solubility and IMF were correlated between BF and LTL and between TB and LTL.

**Table 5.** Correlation coefficient (Pearson’s *r*) of texture and chemical composition between *Longissimus thoracis et lumborum* (LTL) and *Biceps femoris* (BF) or *Triceps Brachii* (TB)

	LTL-BF	LTL-TB
Cooking loss (%)	0.42***	
WBSF (N)	0.00	
Hardness (N)	0.30*	
Cohesiveness	0.01	
Adhesiveness (Nmm)	0.00	
Chewiness (N)	0.23	
Resilience	−0.07	
Springiness	0.15	
pH	0.29*	−0.05
Collagen content (mg/g)	0.30*	0.15
Collagen solubility (%)	0.44***	0.32*
IMF (%)	0.57***	0.57***

*P < 0.05, **P < 0.01, ***P < 0.001.

WBSF, Warner–Bratzler shear force; IMF, intramuscular fat.

## Discussion

The major findings of this study were that 1) BF was tougher than LTL in TPA measurement; 2) TB showed the highest pH, collagen, and IMF content; 3) pork from winter was tougher than that from summer; 4) sex and weight group did not affect texture of pork but pork from castrates had higher collagen solubility than female pork; 5) pH, collagen solubility, and IMF were related to pork texture in both muscles; 6) the texture and chemical component of BF and TB were correlated with some properties of LTL. Therefore, hypothesis 1) was accepted and hypothesis 3), 4), and 5) were partly accepted, while hypothesis 2) was rejected.

There were distinct differences between muscles in objective measurements. In this study, BF showed higher cooking loss and was tougher than the LTL, which was similar to previously reported results (Hwang et al., 2004; Li et al., 2022a). pH of TB was the highest, possibly because of its high density of type I fibre, enzyme activities, and ATP concentration (Uhrín and Liptaj, 1992; Choe et al., 2008; Krischek et al., 2019). Collagen content was the highest in TB and the lowest in LTL. The muscle difference in collagen content is due to their functions in locomotion (Dubost et al., 2013). During physical movement, connective tissues facilitate force transmission and the functional efforts of locomotive muscle, such as BF and TB, are greater than those of positional muscle, such as LTL (Purslow, 2010). Therefore, different extent of physical movement could cause different collagen content among muscles.

In this study, the IMF content was the highest in TB, and there was no difference between BF and LTL. In previous studies, the IMF of TB was higher than those of LTL, although the comparison of IMF content between these 3 muscles varied. Essien (1988) reported that IMF content was in the order of BF > TB > LTL, while Kim et al. (2008) found that the IMF content of BF and TB were similar and they were higher than that of LTL. However, in our previous study, the IMF content of pork BF and LTL were similar (Li et al., 2022a). The differences in IMF between muscles result from oxidative and glycolytic metabolism in muscles and slaughter weight relative to sexual maturity (Hocquette et al., 2010). Muscles with more oxidative fibre tend to have more IMF (Hocquette et al., 2010). The TB contains higher density of oxidative fibre than the LTL and BF. Therefore, the IMF content of TB is higher than the other 2 muscles (Franck et al., 2007), while the lower IMF content in BF in the present study was caused by the genetic variations between studies (Hocquette et al., 2010).

Season influenced pork quality. Winter pork was likely tougher than that from summer, with higher collagen solubility, which was different from what we expected. Gajana et al. (2013) and Hernández-Álvareza et al. (2015) also reported that pork LTL from winter had higher WBSF than that from summer. Van de Perre et al. (2010) found that pH of winter pork *gracilis*, SM, and LTL tended to be lower than that of the summer samples and that summer samples had a higher incidence of pale, soft, and exudative (PSE). The higher collagen solubility in winter pork might be caused by higher daily feed intake and energy intake due to low temperature (Hale and Johnson, 1970). An increase in energy intake enhances collagen turnover and collagen synthesis, which might promote enzyme activity in healthy pigs, leading to a higher proportion of newly synthesized and soluble collagen (Cranwell et al., 1996). However, higher collagen solubility is usually related to higher tenderness (Girard et al., 2012). The effects of season on pork tenderness and collagen characteristics need further investigation.

Castrated pork showed higher collagen solubility than female pork. This finding was similar to a previous study (Chaosap et al., 2018), although no difference between the 2 sexes has also been reported (Aaslyng et al., 2018; dos Santos et al., 2021). Intact male animals have higher serum testosterone, which promotes collagen synthesis at an early age, leading to more newly synthesized collagen and higher collagen solubility than castrated male (Gerrard et al., 1987; Miller et al., 1989). However, when comparing castrated male and female pork, there was no definite answer. More studies are required to find out the mechanism of higher collagen solubility in castrated male compared to female pork.

Ultimate pH was negatively related to pork cooking loss and instrumental tenderness in the present study. In this study, pH was measured after freeze-thawing, but meat was cooked from frozen, and the pH measurement was in accordance with this situation. Previous studies also reported that pH was negatively correlated with pork cooking loss and WBSF (Huff-Lonergan et al., 2002; Lonergan et al., 2007; Jeleníková et al., 2008). Enzymes, such as calpain, contributes to post-mortem proteolysis, resulting in the weakening of myofibrillar structure and tenderization of meat. The activity of these enzymes is affected by muscle pH (Lomiwes et al., 2014). Also, when muscle pH is higher than the isoelectric point, proteins have net charge, allowing proteins to repel each other and increasing water-holding capacity (Huff-Lonergan and Lonergan, 2005). Therefore, pork with higher ultimate pH is tenderer and more juicy. In the present study, pH was not correlated with cooking loss in both muscles, but they were correlated in individual muscles (Table 3). However, when other factors such as sex and weight group were added to the model, their relationships were significant (Table 4). pH varied among animal factors, which play an important role in the contribution of pH to water-holding capacity. Also, the effects of ultimate pH on pork texture were different in the LTL and BF, indicating that the contribution of pH to pork texture differ between muscles.

In this study, IMF was negatively related to pork chewiness, hardness, WBSF, and cooking loss, only in the LTL but not in the BF. The IMF is generally considered to influence tenderness, juiciness, and flavour of meat, but there are contradictory findings (Hocquette et al., 2010). Smith and Carpenter (1976) proposed that a higher IMF content reduced protein content, and cooked fat was easier to shear than myofibrillar proteins (Warner et al., 2021). However, the effects of IMF on pork texture also depend on some confounding factors, such as breed (Laack et al., 2001) and pH (Lonergan et al., 2007). Breed affected IMF content in pork and might then affected texture. In the present study, the effect of IMF on pork texture was likely to be driven by its effect in LTL, even though BF and LTL did not differ in IMF content. Nevertheless, IMF was not correlated with WBSF in either muscle, which might be caused by the indifferent IMF content and WBSF between BF and LTL. Caine et al.(2003) reported that TPA was a better predictor for sensory tenderness than WBSF. Therefore, TPA could differentiate BF and LTL and thus showed significant relationship with IMF. With the low range of IMF in BF and LTL, there is a negative relationship between IMF and pork texture, and the relationship varied between muscles.

Collagen solubility was related to hardness and WBSF in both muscles and to chewiness in BF in this study. The effects of collagen solubility on meat texture varied in previous studies (Ngapo et al., 2002; Christensen et al., 2011; Girard et al., 2012). A meta-analysis on beef showed that collagen solubility was negatively correlated with WBSF, especially in muscles other than the loin (Li et al., 2022b). Purslow (2014) proposed that there are 2 pools of collagen, one pool easily degraded by heat and the other resistant to heat and ageing. It is the pool of collagen which is resistant to heat that contributes to cooked meat toughness (Purslow, 2014). The LTL had lower collagen content and less heat-resistant collagen than BF. As a result, the effect of collagen solubility on pork texture was more significant in the BF.

Predicting the quality of different pork muscles from LTL is often of interest to the industry. In the present study, the IMF of BF and TB was moderately correlated with that of LTL. Similarly, Font-i-Furnols et al. (2019) reported that IMF of pork LTL, BF and *gluteus medius* were correlated, with Pearson’s *r* between 0.53 and 0.70. However, Ramírez and Cava (2007) failed to find a significant correlation between IMF content of BF and LTL. It is possible that the increase of IMF content is consistent for all muscles, but the current results did not support the prediction of IMF content of BF and TB using IMF of LTL.

## Conclusion

Pork quality is affected by muscle, season, sex, and carcass weight. Pork BF was tougher than the LTL, and pork from winter was tougher than summer samples. TB had the highest pH, collagen and IMF content. pH, collagen solubility, and IMF of pork LTL and BF were related to pork texture and cooking loss, but their relationships differed between muscles. Different muscles must be considered when investigating the effects of chemical components on pork quality. There were some significant correlations in pork quality between BF, TB, and LTL, but they were not good enough to build prediction models for the quality of BF and TB using the data of LTL. Further studies can investigate approaches to manipulate muscle, season, sex, and carcass weight to achieve better pork quality.

## Abbreviations

BF: *Biceps femoris*
IMF: intramuscular fat
LTL: *Longissimus thoracis et lumborum*
PC: principal component
PCA: principal component analysis
SM: semimembranosus
TB: *Triceps brachii*
TPA: texture profile analysis
WBSF: Warner–Bratzler shear force
